# DNA methylation risk score for type 2 diabetes is associated with gestational diabetes

**DOI:** 10.1186/s12933-024-02151-z

**Published:** 2024-02-13

**Authors:** Teresa M. Linares-Pineda, Nicolas Fragoso-Bargas, María José Picón, Maria Molina-Vega, Anne Karen Jenum, Line Sletner, Sindre Lee-Ødegård, Julia O. Opsahl, Gunn-Helen Moen, Elisabeth Qvigstad, Rashmi B. Prasad, Kåre I. Birkeland, Sonsoles Morcillo, Christine Sommer

**Affiliations:** 1 Department of Endocrinology and Nutrition, Instituto de Investigación Biomédica Málaga (IBIMA)- Plataforma Bionand, University Hospital Virgen de la Victoria, Málaga, Spain; 2 Department of Biochemistry and Molecular Biology 2, University of Granada, Granada, Spain; 3 Department of Endocrinology, Morbid Obesity and Preventive Medicine, Oslo University Hospital, Oslo, 0424 Norway; 4 Institute of Clinical Medicine, Faculty of Medicine, University of Oslo, Oslo, Norway; 5 Mohn Center for Diabetes Precision Medicine, Department of Clinical Science, University of Bergen, Bergen, Norway; 6 General Practice Research Unit (AFE), Department of General Practice, Institute of Health and Society, University of Oslo, Oslo, Norway; 7 Department of Pediatric and Adolescents Medicine, Akershus University Hospital, Lørenskog, Norway; 8 Haukeland University Hospital, Bergen, Norway; 9 Institute of Molecular Bioscience, The University of Queensland, Brisbane, Australia; 10 K. G Jebsen Center for Genetic Epidemiology, Department of Public Health and Nursing, NTNU, Norwegian University of Science and Technology, Trondheim, Norway; 11 Population Health Science, Bristol Medical School, University of Bristol, Bristol, UK; 12 Frazer Institute, The University of Queensland, Woolloongabba, QLD 4102 Australia; 13 Lund University Diabetes Centre, Malmo, Sweden; 14 Institute for Molecular Medicine Finland (FIMM), University of Helsinki, Helsinki, Finland; 15 Centre for Biomedical Research Network on Obesity Physiopathology and Nutrition (CIBEROBN), Madrid, Spain

**Keywords:** Gestational diabetes, Methylation risk score, Type 2 diabetes, Epigenetics, DNA epigenetics

## Abstract

**Background:**

Gestational diabetes mellitus (GDM) and type 2 diabetes mellitus (T2DM) share many pathophysiological factors including genetics, but whether epigenetic marks are shared is unknown. We aimed to test whether a DNA methylation risk score (MRS) for T2DM was associated with GDM across ancestry and GDM criteria.

**Methods:**

In two independent pregnancy cohorts, EPIPREG (*n* = 480) and EPIDG (*n* = 32), DNA methylation in peripheral blood leukocytes was measured at a gestational age of 28 ± 2. We constructed an MRS in EPIPREG and EPIDG based on CpG hits from a published epigenome-wide association study (EWAS) of T2DM.

**Results:**

With mixed models logistic regression of EPIPREG and EPIDG, MRS for T2DM was associated with GDM: odd ratio (OR)[95% CI]: 1.3 [1.1–1.8], *P* = 0.002 for the unadjusted model, and 1.4 [1.1–1.7], *P* = 0.00014 for a model adjusted by age, pre-pregnant BMI, family history of diabetes and smoking status. Also, we found 6 CpGs through a meta-analysis (cg14020176, cg22650271, cg14870271, cg27243685, cg06378491, cg25130381) associated with GDM, and some of their methylation quantitative loci (mQTLs) were related to T2DM and GDM.

**Conclusion:**

For the first time, we show that DNA methylation marks for T2DM are also associated with GDM, suggesting shared epigenetic mechanisms between GDM and T2DM.

**Supplementary Information:**

The online version contains supplementary material available at 10.1186/s12933-024-02151-z.

## Introduction

Type 2 Diabetes Mellitus (T2DM) affects 10.5% of the global population in the age group 20 to 79 years. T2DM threatens health of the individuals and healthcare systems due to its numerous complications and high healthcare cost and is among the top ten causes of death in the world [1].

During pregnancy, insulin resistance increases to maintain adequate glucose flow to the offspring. However, if the pancreatic beta cells cannot compensate with sufficiently high insulin secretion, this can result in hyperglycaemia and Gestational Diabetes Mellitus (GDM), which is defined by hyperglycaemia with first onset during pregnancy [2]. GDM increases the risk of pregnancy complications such as pre-eclampsia, caesarean section, neonatal hypoglycaemia, preterm birth, and foetal macrosomia. Moreover, GDM severely increases the future T2DM risk in the mother [3]. Although the prevalence of GDM is increasing globally, it varies depending on population characteristics and the diagnostic criteria used [4].

Epigenetics is the study of changes in gene expression that are not caused by changes in the DNA sequence. Some epigenome-wide association studies (EWAS) suggest that epigenetic mechanisms contribute to the pathogenesis of T2DM [5–7]. Genetic risk scores (GRS) have been increasingly used to assess disease risk, such as for T2DM [8, 9]. Similarly, methylation risk scores (MRS) are increasingly studied, to assess associations with outcomes of interest such as some cancers and kidney disease [10], and show promising potential as a tool to aid the prediction of T2DM and to understand gene-environment interactions. Schrader et al. showed that MRS separated T2DM subjects into different groups and were associated with diabetic complications like cardiovascular disease, chronic kidney disease and retinopathy [11].

Despite similar genetics and pathophysiology between GDM and T2DM [12], genetics only explain a small proportion of overall T2DM risk, and environment is known to play an important role for epigenetics. However, whether epigenetic marks are common between GDM and T2D has not been reported previously. Epigenetics marks common for GDM and T2DM may help us understand these pathological mechanisms better in order to prevent, diagnose or improve treatment of GDM and T2DM. We aimed to test whether an MRS for T2DM is associated with a GDM across ancestry and GDM criteria.

## Methods

### Study population

#### The EPIPREG cohort

A subgroup of 480 women was selected from the STORK Groruddalen (STORK G) pregnancy cohort. STORK G included 823 healthy women from different ethnic origins (European, South Asian, African, Middle Eastern and South American) who attended three different Child Health Clinics in the area of Groruddalen, Oslo, Norway, during the 2008–2010 period [13]. Ethnic origin was defined by either the individual’s country of birth or their mother’s country of birth if the latter was born outside of Europe [17]. The EPIPREG subgroup has a total of 312 European subjects (EPIPREG_EU), whereof 73 were diagnosed with GDM, and 168 South Asians (EPIPREG_SA), whereof 68 were diagnosed with GDM. European and South Asian ancestry was determined by genetic principal components [14]. Fasting blood samples were collected, and a 75 g oral glucose tolerance test (OGTT) was offered to all women (universal testing) at week 28 ± 2 of pregnancy. For the present study GDM was classified according to the slightly modified International Association of the Diabetes and Pregnancy (IADPSG) criteria (fasting glucose ≥ 5.1 mmol/l and/or 2-hour glucose ≥ 8.5 mmol/l, as 1-h glucose values were not available).

The Norwegian Regional Committee for Medical Health Research Ethics South East approved the STORK-G study including genetic and epigenetic data (ref. number 2015/1035). Written informed consent from all participants was obtained before any study-related procedure.

#### EPIDG cohort

A total of 32 (16 GDM, 16 non-GDM) pregnant women were selected from EPIDG cohort, which started in 2019 and is still recruiting participants. The EPIDG cohort has a total of 230 Mediterranean (South European) pregnant women who attended the Unit of Diabetes and Pregnancy at University Hospital Virgen de la Victoria, Málaga, Spain. All participants gave their consent to participate in the study. GDM criteria followed a two-step strategy from the National Diabetes Data Group (NDDG) guideline [15]. The first step was a screening using the O’Sullivan test (50 g glucose overload) between 24 and 28 weeks. Then an oral glucose tolerance test (OGTT)-100gr was performed on those women with a positive result in the O’Sullivan test (≥ 0.7.8 mmol/L). GDM was diagnosed if glucose values were for the OGTT-100 higher than the threshold, at least at two points: fasting > 5.8 mmol/l, after 1 h > 10.6 mmol/l, after 2 h > 9.2 mmol/L, after 3 h > 8.0mmol/l. Pregnant women with normal OGTT-100gr were considered as controls (non-GDM). The 32 samples were selected based on the availability of blood samples and matched by age, gestational age and pre-pregnant BMI. The study was approved by the Institutional review board at the Hospital Universitario Virgen de la Victoria, Málaga.

### Samples extraction, DNA isolation and bisulfite conversion

In both cohorts, samples were collected in gestational week 28 ± 2. The samples were either aliquoted and biobanked or subject to routine laboratory analyses. In the EPIPREG sample, DNA from peripheral blood leukocytes was extracted subsequently throughout the data collection, at the Hormone Laboratory, Oslo University Hospital, using a salting out procedure [16], and stored at -80ºC. EZ DNA methylation TM Kit (ZYMO Research, Tustin, CA, USA) was used for the bisulphite conversion of DNA. In EPIDG cohort, DNA from peripheral blood leukocytes was extracted using DNA Blood Mini Kit (Quiagen, Hiden, Germany). Epitect Bisulfite Kit (Qiagen, Germany) was used for bisulphite conversion. DNA methylation in both cohorts was quantified with Infinium MethylationEPIC BeadChip (Illumina, San Diego, USA).

### Methylation values extraction

Raw data from both studies were analysed in R. In EPIPREG, the Meffil R package (https://cran.r-project.org/) was used for quality control (QC), normalization and reporting of B-values [14]. During the QC procedure implemented in Meffil R package, we removed six samples who were considered outliers from the methylated to unmethylated ratio comparison (> 3SD), one sample that was an outlier in control probes bisulfite 1 and 2 (> 5SD), and one sample due to sex discrepancy (predicted sex outlier > 5 SD). Probes with a bead count fewer than three and dectection *p*-value < 0.01. In total 472 of the 480 samples and 864,560 probes passed the QC. Functional normalization was implemented in Meffil R, which takes into account potential batch effects such as slide, row and columns. Proportions of blood cells (namely, CD8T, CD4T, NK, β-cells, monocytes, and neutrophils) were calculated using the Houseman method [27] during the QC [17].

After processing, 472 of the 480 available individuals remained and 810.266 in the EPIPREG sample passed QC. In EPIDG, the full sample and 741.479 probes passed QC (Supplementary Fig. 1).

### Methylation risk score

**Fig. 1:**
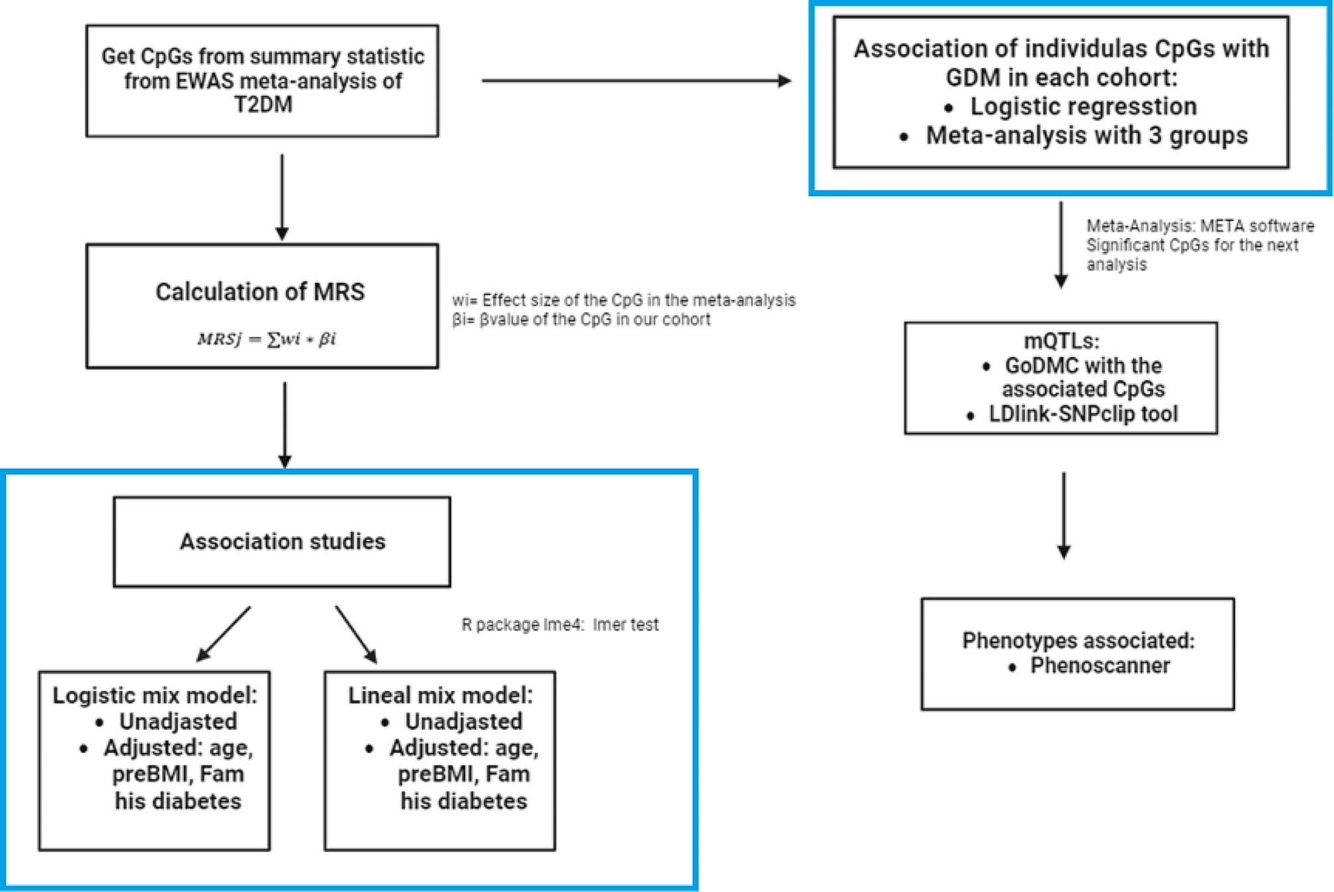
Workflow of the methodology followed to construct the MRS and the rest of the statistical analysis. preBMI: previous pregnancy BMI, Fam his diabetes: family history of diabetes. Blue Line indicate the in-house data vs. the public data used in our study

To construct the weighted DNA MRS for T2DM in our sample we used summary data for the CpGs sites discovered in an EWAS of T2DM in five prospective European cohorts (*n* = 5.859) [7, 18–21]. The regression coefficients for the EWAS of T2DM was based in β-values. We calculated MRS for each of the discovered CpG sites available in our two cohorts. Due the QC filters of CpGs in each cohort only 42 of 72 CpG could be included in the MRS (Supplementary Table 1). MRS was constructed by multiplying the regression coefficient from the EWAS of T2DM with individual β-values in EPIPREG and EPIDG (EPIPREG-EU, EPIPREG-SA and EPIDG). Thereafter we summarized the individual score for each of the 42 CpGs sites to obtain the MRS. Figure 1 illustrates the workflow, including development of the MRS and the statistical analysis.

### Statistical analysis

All the statistical analyses were performed using Rstudio (3.4.4). Clinical variables were compared between GDM and non-GDM in each group separately (EPIPRE_EU, EPIPREG_SA and EPIDG), depending on if the variable was normally distributed or not, t-test or Whitney U test were used for the continuous variable. A logistic mixed model regression was used to elucidate if MRS was associated with GDM in our three groups. Linear mixed model regressions were performed to test the association between MRS and continuous variables such as HOMA-IR, C-peptide, fasting glucose and 2 h glucose level, using a Bonferroni corrected threshold *p* < 0.0125 (0.05/4). We used the R packages lme4 and lmerTest [22] to perform mixed models regression. In these models, ancestry (European, South Asian, Mediterranean (EPIDG)) was treated as a random intercept to overcome potential ancestry-related differences in DNA methylation. We performed an unadjusted and an adjusted model by age, pre-pregnant BMI, family history of diabetes and smoking status, since these are covariates that are associated with both epigenetic marks and gestational diabetes.

To identify individual CpG sites associated with GDM in the studied populations, we performed logistic regressions for each CpGs used in the MRS across the three groups, followed by a fixed effects meta-analysis of the three groups for each CpG site using METAL [23]. Since random-effect models may bias the results if smaller studies have a large effect compared to well-performed, larger studies [24], we decided to use a fixed-effect model for a conservative approach, where the case-control design in EPIDG would not count more than the population-based design in EPIPREG.

### mQTLs and phenotypes associated

Due to low statistical power for mQTL analysis in EPIPREG, we performed look-ups in GoDMC of the significant CpGs obtained in the meta-analysis to identify methylation quantitative trait loci (mQTL) to see if these methylation sites were influenced by genetic variants. GoDMC include Cis and trans meta-analysis results from genome-wide scans of 420.509 DNA methylation sites. This information come from several projects, with the aim to share data on genetic basis of DNA methylation variation. GoDMC provides a list of SNPs that have been associated with the CpG of interest. We used the LDlink-SNPclip tool [25] to identify SNPs in linkage disequilibrium (r^2^ > 0.8, MAF = 0.01) and the selected populations were European and South Asians. To assess relevance of the mQTLs to diabetes related phenotypes, we performed explorative look-ups in Phenoscanner [26] of phenotypes nominally associated with the mQTLs. Phenoscanner is a database holding publicly available results from large-scale GWAS. Although the default threshold used by Phenoscanner is *p* < 1 × 10^− 5^, we also looked up phenotypes with *p*-value < 0.05 due to the explorative nature of this look-up [27].

## Results

### Characteristics of the samples

The clinical characteristics of EPIPREG and EPIDG are represented in Table 1. In the EPIPREG cohort, non-GDM and GDM women differed significantly in pre-pregnant BMI, fasting glucose (Gluc0), 2 h glucose after OGTT (Gluc2), systolic blood pressure (SBP), glycosylated hemoglobin A1C (HbA1C), Homeostatic Model Assessment for insulin resistance (HOMA-IR), triglycerides (TAG), cholesterol and HDL across ethnicities. Only fasting glucose levels differed significantly between GDM cases and controls in the EPIDG cohort (Table 1).

**Table 1:** Characteristics of the study subjects in each cohort

	EPIPREG						EPIDG		
Cohorts	European *n* = 312			South Asian *n* = 168					
Variable	non-GDM (*n* = 237)	GDM (*n* = 73)	***P***.val	non-GDM (*n* = 99)	GDM (*n* = 69)	***P***.val	Non-GDM (*N* = 16)	GDM (*N* = 16)	***P***-value
**Age**	30.08 (4.47)	30.14 (4.95)	0.9421	27.87(4.512)	28.69(4.68)	0.2661	34.1(4.5)	33.81(4.1)	0.808
**Gestational age (weeks)**	28.10 (12.5)	28.04(13.83)	0.716	27.5 (10.2)	27.8(12.05)	0.194	27.56(2.1)	28.06 ± 2.8	0.579
**O’Sullivan (mg/dl)**							160.7(16.3)	172.94(22.2)	NS
**Fasting glucose (mmol/l)**	4.48 (0.32)	5.461 (0.59)	**2.2E-16**	4.57(0.322)	5.5(0.526)	**2.2E-16**	4.55(0.4)	4.96(0.6)	**0.036**
**2 h gGlucose (mmol/l)**	5.7(1.11)	7.038 (0.98)	**3.9E-09**	5.94(0.451)	7.064(0.542)	**6.05E-06**			
**prepregnant BMI (kg/m** ^**2**^ **)**	24.077 (5.01)	26.27 (6.05)	**0.00503**	23.004(3.215	24.84(4.446)	**0.0064**	25.5(4.19)	25.8(4.5)	0.862
**SBP (mmHg)**	105.88(9.46)	110.07(9.19)	**0.001**	99.24(8.53)	103.94(8.25)	**0.001**	104.25(9.9)	111.4(15.4)	0.131
**DBP (mmHg)**	67.81(6.99)	69.68(7.22)	**0.027**	65.48(7.68)	67.08(5.78)	0.075	69.1(8.2)	70.1(7.8)	0.71
**HbA1C (%)**	5.037 (0.255)	5.22(0.338)	**4.3E-05**	5.19(0.313)	5.4 (0.333)	**5.31E-05**	5.1(0.28)	5.3(0.37)	0.151
**HOMA-IR**	1.55(1.12)	1.936(0.922)	**0.0014**	1.694(0.678)	2.277(0.512)	**6.02E-06**	1.6(0.7)	2.1(1.2)	0.147
**TAG (mmol/l)**	1.94 (0.721)	1.969(0.68)	0.82	1.997(0.583)	2.08(0.681)	0.42	2.2(0.6)	2.2(0.5)	0.829
**Total cholesterol (mmol/l)**	6.51(1.093)	6.016(1.05)	**0.0007**	6.186(1.025)	5.753(1.027)	**0.0084**	7.04(1.2)	6.7(1.3)	0.446
**HDL (mmol/l)**	1.97 (0.412)	1.835(0.408)	**0.0112**	1.941(0.436)	1.80(0.451)	**0.0467**	2.1(0.47)	2.01(0.4)	0.424
**Smoking (Yes/No)**	71/45	28/45	**0.046**	2/95	1/67	**1.21E-15**	0/16	0/16	0.310

BMI: body mass index. SBP: systolic blood pressure. DBP: diastolic blood pressure. HDL: high density lipoprotein cholesterol. TAG: triacylglycerol. GDM: Gestational Diabetes Mellitus group. Non-GDM: non- Gestational Diabetes Mellitus group

### Methylation risk score

**Fig. 2:**
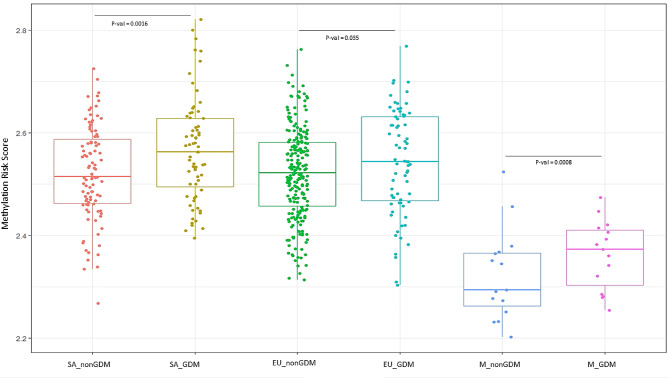
Boxplot MRS for each group: red (SA_nonGDM)- EPIPREG south Asians non-GDM, Yellow (SA_GDM)- EPIPREG South Asians GDM, Green (EU_nonGDM)- EPIPREG Europeans non-GDM, Light Blue (EU_GDM): EPIPREG Europeans GDM, Dark Blue (M_nonGDM)- EPIDG (Mediterraneas) non-GDM, Pink (M_GDM)- EPIDG GDM

In the mixed models regression, MRS for T2D levels were higher in women with GDM compared to non-GDM (adjusted model O.R: 1.4, 95%C.I: 1.10–1.74, P.val = 0.00014). For continuous traits, the MRS for T2D was significantly associated only with fasting glucose in the adjusted linear mixed models (P.val = 0.0086) (Table 2). When assessing EPIDG, EPIPREG Europeans and EPIPREG South Asians separately, MRS for T2D was associated with higher OR for GDM in EPIPREG South Asians, and followed the same direction of effect in EPIPREG Europeans and EPIDG although not statistically significant (Fig. 2; Supplementary Table 2).

### mQTLs for CpG sites common between T2DM and GDM

In the meta-analysis of the 42 CpG sites included in the MRS for T2DM, six CpGs were significantly associated with GDM (FDR < 0.05) (Table 3). All the identified CpG sites, except cg25130381, had significant mQTLs from lookups in GoDMC. A total of 23 mQTLs were found (supplementary Table 3). According to lookups in Phenoscanner, only two CpGs had mQTLs associated at *p*-value < 1 × 10^− 5^ with phenotypes related to immunoglobulin G, Rheumatoid arthritis, and body composition (Supplementary Table 4). However, when using a liberal threshold (*p*-value < 0.05), we observed some mQTLs possibly related to diabetes related phenotypes (Table 4).

**Table 2:** Association between the MRS for T2D and phenotypes in EPIPREG and EPIDG

	Model	Estimate	Std.Error	df	***t***.value	***P***.val
HOMA-IR	Unadjusted	0.989	0.802	0.0479	1.23	2.18E-01
C-peptide		0.422	0.167	0.0483	2.535	**0.0116**
Fasting glucose		0.8351	0.2726	0.0279	3.064	**0.0024**
2 h glucose		0.751	0.707	0.451	1.063	0.2882
HOMA-IR	Adjusted*	0.416	0.777	0.0468	0.536	0.592
C-peptide		0.254	0.152	0.0478	1.67	0.0964
Fasting glucose		0.707	0.268	0.0313	2.641	**0.00869**
2 h glucose		0.389	0.701	0.512	0.568	0.57

HOMA-IR: Homeostatic Model Assessment of Insulin Resistance. Gluc.0: fasting glucose; 2h Gluc: glucose level after 2h from the OGTT. *Model adjusted for age, prepregnant BMI, family history of diabetes and smoking status

**Table 3:** List of the 6 CpGs significantly associated with GDM across the three samples (EPIPREG_EU, EPIPREG_SA, EPIDG)

CpG*	Effect	StdErr	Direction	FDR	Genes
cg14020176	1364.7387	334.8719	+++	0.00192906	SLC9A3R1
cg22650271	2288.3955	612.9638	+++	0.003969	SYNGR1
cg14870271	10.0163	2.7782	+++	0.0043652	LGALS3BP
cg27243685	2434.2168	716.9641	+++	0.00576156	ABCG1
cg06378491	3200.9735	938.6366	+++	0.00576156	MAP4K2
cg25130381	106,813,769	38646.6933	+++	0.039984	SLC9A1

*This model was performed using β-values

**Table 4:** mQTLs and disease associated, according to phenoscanner

ID_cg	Gene of cg	Chr	Position	rs		Gene of rs	Chr	Position		A1	A2	Disease	Beta	***P***-val
cg06378491	MAP4K2	11	64,564,012	rs74374453		AP005273.1	11	64,271,540	cis	A	G	Insulin-like growth factor 1		4.96E-02
												**Gestational diabetes only**	-0.086	2.99E-03
												Self-reported type 1 diabetes	-0.0007011	4.28E-02
cg14020176	SLC9A3R1	17	72,764,985	rs2385067		TMEM104	17	72,810,070	cis	A	G	**Type II diabetes**	-0.195	4.62E-04
				rs652963		QSER1	11	32,911,737	trans	T	C	Illnesses of siblings: diabetes	-0.002764	7.23E-03
												**Type II diabetes**	-0.036	2.80E-02
cg14870271	LGALS3BP	17	76,976,010	rs117549034		USP36	17	76,826,416	cis	C	T	Parkinson Disease	-0.02651	2.82E-06
												**Diabetes mellitus in pregnancy**	-0.0004308	1.18E-02
												Started insulin within one year diagnosis of diabetes	0.04261	1.40E-02
												Eye problems or disorders: diabetes related eye disease	-0.005733	4.11E-02
cg22650271	SYNGR1	22	39,760,165	rs5757582		AL031590.1	22	39,661,032	cis	A	C	Rheumatoid arthritis	-0.06188	1.90E-05
												Crohn’s disease	0.000472	4.76E-04
												**Type II diabetes adjusted for BMI**	0.038	9.40E-03
				rs7289325		PDGFB		39,642,577	cis	A	T	Started insulin within one year diagnosis of diabetes	-0.009563	1.25E-02
												**Gestational diabetes only**	0.01211	4.44E-02
				rs742402		AL031590.1		39,659,487	cis	A	G	High cholesterol	-0.002935	4.90E-04
												Crohns disease	-0.0004341	2.30E-03
												Rheumatoid arthritis	0.06766	1.70E-05
												**Type II diabetes adjusted for BMI**	-0.04	9.50E-03
												Illnesses of mother: diabetes	-0.001557	4.31E-02
												Medication for cholesterol, blood pressure or diabetes: none of the above	0.003574	4.64E-02
				rs9611137		SCUBE1		39,682,445	cis	C	T	Crohn’s disease	-0.2224	3.76E-05
												Inflammatory bowel disease	-0.152	8.48E-05
												High cholesterol	-0.00366	2.90E-02
												Medication for cholesterol, blood pressure or diabetes: cholesterol lowering medication	-0.00794	1.33E-02
												**Type II diabetes adjusted for BMI**	-0.071	2.90E-02

## Discussion

This is the first study to show that an MRS for T2DM is associated with GDM across our two cohorts, diagnostic criteria and ancestry. In addition, the MRS for T2DM was associated with higher fasting glucose levels. Six CpGs of the 42 included in the MRS, were significantly associated with GDM across EPIPREG Europeans, EPIPREG South Asians and EPIDG Mediterranean. We identified 23 mQTLs linked to the 6 CpG sites. Some of them were associated with T2DM and GDM with nominal significance in lookups of GWAS summary data.

GDM and T2DM share many pathophysiological factors, but the exact underlying mechanisms are largely unknown. There is a need to know how T2DM and GDM are linked to improve the prevention and thus avoid metabolic complications in the future. Genome-wide association studies (GWAS) have been used to investigate the potential link between GDM and T2DM [12], but none have so far tested whether GDM and T2DM share epigenetic marks. An increasing number of studies suggest that environmental factors are associated with epigenetic marks and that those marks may be important to understand interactions between genetics and environmental factors. A few EWAS of GDM in maternal peripheral blood are published so far, but the data is not sufficient for an MRS for GDM due to heterogeneity in diagnosis criteria used as well as their small sample sizes [28–31].

Our MRS was constructed from a meta-analysis of five European cohorts that identified CpGs associated with incident T2DM [18]. According to the bibliography of the genes included in the MRS, some of them were associated with glucose metabolism [32, 33], T2DM, type 1 diabetes mellitus and GDM [34]. ATP binding cassette subfamily G member 1 (*ABCG1*) is a gene involved in macrophage cholesterol and phospholipid transport and may regulate cellular lipid homeostasis. *ABCG1* has been associated with diabetes and metabolic syndrome [35]. Furthermore, some studies show that GDM affects cholesterol homeostasis through this gene, therefore it could have a role in GDM and cardiovascular events [36].

The mQTLs were nominally associated with T2DM and GDM in lookups, but were interestingly also associated with phenotypes related to body composition, immunoglobulin G and autoimmune diseases associated with inflammation, such as rheumatoid arthritis and Crohn’s disease. These observations may suggest a pleiotropic relationship between these mQTLs for diabetes related CpG sites, but further exploration is necessary to assess the relevance and understand potential mechanisms.

The strengths of this study include the combination of two independent cohorts with case-control design in EPIDG which maximizes differences in GDM and non-GDM versus the population-based design in EPIPREG which includes the full range of values including less severe GDM. This combined with the two ancestries in EPIPREG, strengthen the evidence that these CpG sites may be important to understand the common epigenetic grounds of GDM and T2D. Limitations include limited statistical power in analysis of each sample separately and in associations between separate CpG sites and GDM. Still, the MRS for T2D was associated with GDM in EPIPREG South Asians, with the same direction of effect in EPIPREG Europeans and EPIDG. Further, an MRS for GDM based on an EWAS of GDM instead could have given a more precise prediction of GDM. Unfortunately published EWAS of GDM are still few and have small sample sizes which would result in a less robust MRS [40–42]. Also, our cross-sectional study design cannot entangle whether GDM status influence DNA methylation or vice versa. Hence this MRS should be tested in early pregnancy to be considered as potential predictor of GDM.

Genetic risk score reflects the inherited risk but have shown poor performance in complex disease such as diabetes. MRS is thought to reflect environmental triggers of the disease or phenotype and help to understand disease mechanism. Recent studies have shown the utility of both GRS and MRS for clinical prediction of different phenotypes [43]. Although GRS is being used in multiple diseases, their use present some limitations [44]. For example, GRS doesn’t reflect the effect of the environment on the phenotype whereas MRS incorporate both genetic factors and environmental exposures and their variation over time. Undoubtedly, combining both scores would enhance their clinical application for predicting or stratification of risk subjects. The MRS level differed between EPIPREG and EPIDG cohort, suggesting that the MRS may be population specific. Although we cannot rule out that part of these differences may be due to the batch or handling effect, they could also be a result of the EPIDG control group not having negative O’sullivan test, while non-GDM women in EPIPREG had normal fasting and 2 h glucose values (1 h glucose OGTT missing in EPIPREG). Further, the populations in EPIPREG and EPIDG differ largely in many aspects, including the severity of GDM.

To conclude, we are the first to show that MRS for T2DM is significantly associated with GDM, suggesting shared epigenetic mechanisms between GDM and T2DM. This may help explain some of the molecular mechanisms mediating the increased risk of developing T2DM after GDM. Future research should compare whether GRS and MRS or a combination of the two provide better predictions of GDM and T2DM and explore how transcription of the identified genes impact methylation on nearby genes. Furthermore, understanding the role of the genetic variants in disease development may help to improve prevention and management of both GDM and T2DM in the future.

### Electronic supplementary material

Below is the link to the electronic supplementary material.

**Supplementary Material 1: Supplementary figure 1.** Work flow for sample selection A) in EPIPREG cohort, and B) EPIDG cohort

**Supplementary Material 2: Supplementary table 1.** List of the 42 CpGs used in MRS from meta-analysis of 5 Europeans cohorts

**Supplementary Material 3: Supplementary table 2.** Logistic model to associate MRS with GDM in each group separately

**Supplementary Material 4: Supplementary table 3.** List of the 5 CpGs with their mQTLS

**Supplementary Material 5: Supplementary table 4.** Phenotypes associated with 2 CpGs with mQTLs higher p-value

## Data Availability

The data of this study are protected by Norwegian data protection law. Data, analytical methods and research materials supporting the results of this study may be reasonably requested from CS and SM.

## Abbreviations

GDM: Gestational Diabetes Mellitus
T2DM: Type 2 Diabetes Mellitus
GRS: Genetic Risk Score
MRS: Methylation Risk Score
OGTT: Oral Glucose Tolerance Test
QC: Quality Control
mQTL: Methylation Quantitative Trait Loci
