# Supplementary figures and images for: DNA methylation risk score for type 2 diabetes is associated with gestational diabetes
Source: Cardiovasc Diabetol. 2024 Feb 13;23:68. doi: 10.1186/s12933-024-02151-z (PMC10865541; doi:10.1186/s12933-024-02151-z)

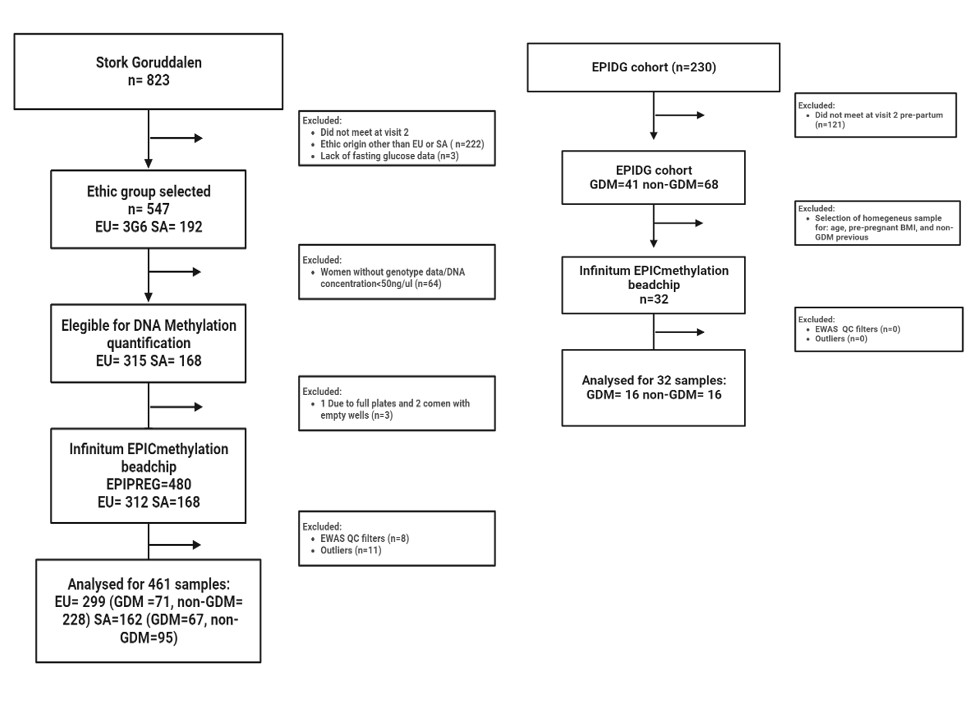

Supplement: Supplementary file 1 — Supplementary Material 1: Supplementary figure 1. Work flow for sample selection A) in EPIPREG cohort, and B) EPIDG cohort [file 12933_2024_2151_MOESM1_ESM.jpg]
