# Supplementary material for: DNA methylation risk score for type 2 diabetes is associated with gestational diabetes
Source: Cardiovasc Diabetol. 2024 Feb 13;23:68. doi: 10.1186/s12933-024-02151-z (PMC10865541; doi:10.1186/s12933-024-02151-z)
Supplement: Supplementary file 2 — Supplementary Material 2: Supplementary table 1. List of the 42 CpGs used in MRS from meta-analysis of 5 Europeans cohorts [file 12933_2024_2151_MOESM2_ESM.docx]

Supplementary Table 1- List of the 42 CpG used in MRS from meta-analysis of 5 Europeans discovery cohorts.

| **Illumina ID** | **Gene name** | **CHR** | **Position** | **Effect size** | **Standard Error** | **P.value** | **FDR** |
| --- | --- | --- | --- | --- | --- | --- | --- |
| cg19693031 | *TXNIP* | 1 | 145441552 | -0.0199 | 0.002 | **3.89E-23** | 1.84E-17 |
| cg06500161 | *ABCG1* | 21 | 43656587 | 0.0109 | 0.0011 | **9.26E-22** | 2.19E-16 |
| cg11024682 | *SREBF1* | 17 | 17730094 | 0.0088 | 0.0011 | **1.41E-14** | 2.23E-09 |
| cg00574958 | *CPT1A* | 11 | 68607622 | -0.0051 | 0.0007 | **6.35E-13** | 7.52E-08 |
| cg05778424 | *AKAP1* | 17 | 55169508 | 0.0075 | 0.0011 | **3.56E-11** | 3.37E-06 |
| cg14020176 | *SLC9A3R1* | 17 | 72764985 | 0.0087 | 0.0014 | **1.53E-10** | 1.21E-05 |
| cg07504977 | *OLMALINC* | 10 | 102131012 | 0.0112 | 0.0018 | **2.17E-10** | 1.47E-05 |
| cg04816311 | *C7orf50* | 7 | 1066650 | 0.0112 | 0.0018 | **2.76E-10** | 1.60E-05 |
| cg14476101 | *PHGDH* | 1 | 120255992 | -0.0145 | 0.0023 | **3.04E-10** | 1.60E-05 |
| cg27243685 | *ABCG1* | 21 | 43642366 | 0.0061 | 0.001 | **4.26E-10** | 2.02E-05 |
| cg14870271 | *LGALS3BP* | 17 | 76976010 | 0.0084 | 0.0014 | **5.00E-10** | 2.15E-05 |
| cg26262157 | *PFKFB3* | 10 | 6214079 | -0.009 | 0.0015 | **1.03E-09** | 3.47E-05 |
| cg18568872 | *ZNF710* | 15 | 90606494 | 0.006 | 0.001 | **1.22E-09** | 3.84E-05 |
| cg01373896 | *KLF16* | 19 | 1854724 | 0.0068 | 0.0011 | **1.30E-09** | 3.84E-05 |
| cg08994060 | *PFKFB3* | 10 | 6214026 | -0.0107 | 0.0018 | **1.66E-09** | 4.46E-05 |
| cg11202345 | *LGALS3BP* | 17 | 76976057 | 0.0078 | 0.0013 | **1.70E-09** | 4.46E-05 |
| cg21480264 | *POLN* | 4 | 2137264 | 0.0059 | 0.001 | **2.45E-09** | 6.12E-05 |
| cg19750657 | *UFM1* | 13 | 38935967 | 0.0089 | 0.0015 | **2.66E-09** | 6.16E-05 |
| cg06192883 | *MYO5C* | 15 | 52554171 | 0.008 | 0.0013 | **2.73E-09** | 6.16E-05 |
| cg06378491 | *MAP4K2* | 11 | 64564012 | 0.0044 | 0.0008 | **3.45E-09** | 7.11E-05 |
| cg11269166 | *METTL8* | 2 | 172203847 | 0.0066 | 0.0011 | **4.19E-09** | 7.94E-05 |
| cg08788930 | *DENND3* | 8 | 142201685 | 0.0073 | 0.0013 | **5.83E-09** | 0.000106 |
| cg22650271 | *SYNGR1* | 22 | 39760165 | 0.0057 | 0.001 | **6.62E-09** | 0.000116 |
| cg03691549 | *TENC1* | 12 | 53443911 | 0.0059 | 0.001 | **9.51E-09** | 0.000161 |
| cg09664445 | *KIAA0664* | 17 | 2612406 | 0.0054 | 0.0009 | **1.03E-08** | 0.000163 |
| cg14956201 | *TRIO* | 5 | 14358153 | 0.0077 | 0.0014 | **1.11E-08** | 0.00017 |
| cg17540192 | *TECPR1* | 7 | 97875259 | 0.0048 | 0.0008 | **1.47E-08** | 0.000218 |
| cg25217710 | *BCAN* | 1 | 156609523 | 0.0052 | 0.0009 | **1.52E-08** | 0.000218 |
| cg16861241 | *FOXJ1* | 17 | 74138396 | 0.0056 | 0.001 | **1.65E-08** | 0.00023 |
| cg15020801 | *PNPO* | 17 | 46022809 | 0.007 | 0.0012 | **1.80E-08** | 0.000244 |
| cg02879453 | *ADCY7* | 16 | 50321818 | 0.0078 | 0.0014 | **3.99E-08** | 0.000525 |
| cg04682775 | *SLC6A9* | 1 | 44495089 | 0.0066 | 0.0012 | **4.17E-08** | 0.000534 |
| cg10639435 | *ZNF250* | 8 | 146104221 | 0.0077 | 0.0014 | **4.28E-08** | 0.000534 |
| cg04927537 | *LGALS3BP* | 17 | 76976091 | 0.0103 | 0.0019 | **4.87E-08** | 0.000592 |
| cg16097041 | *FLAD1* | 1 | 154965544 | 0.0061 | 0.0011 | **5.05E-08** | 0.000599 |
| cg25130381 | *SLC9A1* | 1 | 27440721 | 0.0057 | 0.001 | **5.81E-08** | 0.000671 |
| cg20507228 | *MAN2A2* | 15 | 91460071 | 0.0124 | 0.0023 | **6.37E-08** | 0.000719 |
| cg05460226 | *PIK3R5* | 17 | 8804279 | 0.0095 | 0.0018 | **6.92E-08** | 0.000763 |
| cg01101459 | *LINC01132* | 1 | 234871477 | 0.0065 | 0.0012 | **7.45E-08** | 0.000802 |
| cg25178683 | *LGALS3BP* | 17 | 76976267 | 0.0083 | 0.0015 | **8.06E-08** | 0.000848 |
| cg09072148 | *NRXN2* | 11 | 64491639 | 0.0037 | 0.0007 | **9.11E-08** | 0.000939 |
| cg12322877 | *ASPSCR1* | 17 | 79963213 | 0.0119 | 0.0022 | **9.70E-08** | 0.000978 |

CHR: chromosome, FDR: False discovery rate
