# Supplementary material for: DNA methylation risk score for type 2 diabetes is associated with gestational diabetes
Source: Cardiovasc Diabetol. 2024 Feb 13;23:68. doi: 10.1186/s12933-024-02151-z (PMC10865541; doi:10.1186/s12933-024-02151-z)
Supplement: Supplementary file 4 — Supplementary Material 4: Supplementary table 3. List of the 5 CpGs with their mQTLS [file 12933_2024_2151_MOESM4_ESM.docx]

Supplementary Table 2- List of the 5 CpGs with their mQTLS .

| **ID_cg** | **Gene** | **rs** | **Gene** | **Posicion** | **A1** | **A2** |
| --- | --- | --- | --- | --- | --- | --- |
| cg06378491 | MAP4K2 | rs117645407 | GPR137 | chr22:39748371 | C | T |
|  |  | **rs2375335** | **NRXN2** | **chr11:64490123** | **A** | **G** |
|  |  |  |  |  |  |  |
|  |  | rs630966 | PYGM | chr11:64524911 | C | G |
|  |  |  |  |  |  |  |
|  |  | **rs74374453** | AP005273.1 | chr11:64271540 | A | G |
|  |  |  |  |  |  |  |
|  |  |  |  |  |  |  |
| cg14020176 | SLC9A3R1 | rs174747 | PRRG4 | chr11:32853723 | C | T |
|  |  |  |  |  |  |  |
|  |  | rs2305214 | NAT9 | chr17:72768970 | G | A |
|  |  | rs2384952 | SLC9A3R1 | chr17:72744512 | C | T |
|  |  | **rs2385067** | **TMEM104** | **chr17:72810070** | **A** | **G** |
|  |  | rs492095 | FDXR | chr17:72869078 | G | A |
|  |  |  |  |  |  |  |
|  |  |  |  |  |  |  |
|  |  | **rs652963** | **QSER1** | **chr11:32911737** | **T** | **C** |
|  |  |  |  |  |  |  |
| cg14870271 | LGALS3BP | **rs117549034** | **USP36** | **chr17:76826416** | **C** | **T** |
|  |  |  |  |  |  |  |
|  |  |  |  |  |  |  |
|  |  |  |  |  |  |  |
|  |  | rs35178144 | CYTH1 | chr17:76687205 | A | G |
|  |  |  |  |  |  |  |
|  |  | rs9807193 | ZNF521 | chr18:22818260 | C | T |
|  |  | rs9946154 |  | chr18:22810619 | C | T |
| cg22650271 | SYNGR1 | rs11705183 | SYNGR1 | chr22:39748371 | C | T |
|  |  |  |  |  |  |  |
|  |  | rs17000956 |  | chr22:39679200 | C | T |
|  |  | **rs5757582** | **AL031590.1** | **chr22:39661032** | **A** | **C** |
|  |  |  |  |  |  |  |
|  |  |  |  |  |  |  |
|  |  | rs6001566 | SYNGR1 | chr22:39774448 | A | G |
|  |  |  |  |  |  |  |
|  |  | **rs7289325** | **PDGFB** | **chr22:39642577** | **A** | **T** |
|  |  |  |  |  |  |  |
|  |  | **rs742402** | **AL031590.1** | **chr22:39659487** | **A** | **G** |
|  |  |  |  |  |  |  |
|  |  |  |  |  |  |  |
|  |  |  |  |  |  |  |
|  |  |  |  |  |  |  |
|  |  |  |  |  |  |  |
|  |  | **rs9611137** | **SCUBE1** | **chr22:39682445** | **C** | **T** |
|  |  |  |  |  |  |  |
|  |  |  |  |  |  |  |
|  |  |  |  |  |  |  |
|  |  |  |  |  |  |  |
| cg27243685 | ABCG1 | rs4435094 | SCARB1 | chr12:125351149 | A | G |
|  |  |  |  |  |  |  |
|  |  | rs72497603 | UMODL1 | chr21:43560578 | G | T |
|  |  |  |  |  |  |  |
|  |  |  |  |  |  |  |
|  |  |  |  |  |  |  |
|  |  |  |  |  |  |  |

A1: allele dominant, A2: allele recessive.
